# Mr. Carlisle, on the Use of Bougies

**Published:** 1800-04

**Authors:** Anth. Carlisle

**Affiliations:** Soho-Square


### Medical and Phyfical Journal.

VOL. III.] APRIL, 1800. [NO. XIV.

### To the Editors of the Medical and Phyfical Journal.

GENTLEMEN,

THE general and indifcriminate ufe of bougies, armed with cauftic, and formed of various materials, induces me to trouble you and the public with a few obfervations, in the hope of exciting thofe who have had greater experience, to make a candid declaration of their fuccefs or failure, in the difeafes for which thefe inftruments have been recommended. Mr. J. Hunter's fkill and penetration have not, in all the cafes of his improvements in Surgery, followed the employment of his methods. The ufe of cauftic, which he adopted in cafes of ftrictures in the urethra, was, doubtlefs, often accompanied with fuccefs; but I have reafon to believe that his expectations were, by no means, fo fanguine toward the end of his practice. When novelties are introduced into medicine, whether they be wifely ordered, or the contrary, it feems an invariable confequence, that the hopes of the promulgator are too fanguine; and in the hands of the fanguine imitator, this error is feldom amended.

I do not propofe, in this communication, to fay, that the application of cauftic, in cafes of ftricture in the urethra, has not produced effential benefit to many perfons; but I am called upon by my experience, to ftate thofe inconveniences and dangers which occurred in my own practice. The young furgeon, filled with the ftatements contained in books, and the cures fo eafily performed at the Lecture Table, fets out in the profeffion without fearing the management of any recorded difeafe; he flatters the hopes of the patient, tries his fkill, having, in his own mind, from the firft, a full affurance of fuccefs, and too often meets with the bittereft difappointment. I am aware that a number of great practitioners poffefs more ample materials for the tafk I am undertaking than myfelf; I only hope to draw them forth.

From what I have obferved in the ufe of cauftic to the urethra, I am led to believe, that this ſubſtance ſeldom produces an eſchar, and where it really does ſo, the conſequences are to be highly dreaded. The urethra, defended by its mucus, and capable in every part of throwing out a ſerous fluid, which has a power of diluting the moſt cauſtic ſubſtances, cannot be eaſily acted upon by a cauſtic in its own peculiar quality.

To produce a ſlough or local death in a part by a metallic ſalt, like lunar cauſtic, requires the retention of the ſolvent ſubſtance, in contact with the living part, for a conſiderable length of time, and alſo that the ſolvent ſhould preſerve its concentrated ſtate. None of theſe events happen in the application of cauſtic to the urethra; and, upon ſtrict examination, I have found the ſubſtance voided from the urethra, after the uſe of the lunar cauſtic, to be nothing more than a flake of coagulated mucus, produced by that metallic ſalt. In ſome inſtances, I have ſeen the whole mucus of the urethra thus coagulated, and the power of ſecreting it again thus ſuſpended for ſeveral weeks; the paſſage being moiſtened during that time with a ſerous fluid. When, however, the cauſtic has the effect of producing a ſlough, I fear it will be found to extend far wider than the ſurface touched; and a large part of the urethra will mortify, ſo as to endanger the life of the patient. This calamity, I underſtand, has happened more frequently than is conſiſtent with the unlimited recommendation of ſuch remedies. The effect which appears to me to be deſired from the uſe of cauſtic applications to ſtrictures in the urethra, is that of ulceration; and I believe all the ſucceſsful caſes are cured by the ulcerative proceſs, excited by this irritating ſubſtance, upon a part whoſe powers of action are moderate. Whether any other ſubſtance would produce the ſame effect upon an altered ſtructure, or new formed ſubſtance, like that which conſtitutes a ſtricture, is, I ſuſpect, yet to be tried. The moſt dangerous conſequences which I have witneſſed from the application of lunar cauſtic to ſtrictures, is that of producing an extenſive hæmorrhage, ſuch as to give juſt cauſe for alarm. A young gentleman, to whom I applied the cauſtic for a ſtricture, near the bulbous part of the urethra, had a hæmorrhage produced by it, which continued ſeven days; in the two firſt he loſt four pounds of blood, and nearly as much afterwards. I have heard that ſome perſons have actually died of this kind of hæmorrhage. It would be very honourable to thoſe practitioners, who have ſeen ſuch caſes, to make them public, as it might put the ſurgeon and the patient on their guard. In what manner the lunar cauſtic acts, ſo as to induce hæmorrhages of longer duration than from injuries of the ſame extent, produced duced by cutting or lacerating inftruments, may form a future inquiry. The fact is, however, indifputable. The young practitioner, therefore, availing himfelf of whatever advantages experience may affign to the application of cauftic in ftrictures of the urethra, will do wifely to try other means in fimple cafes; and whenever he feels juftified in reforting to this method, will be apprized of the danger to be apprehended in bad habits, from extenfive floughing of the urethra, and from a degree of hæmorrhage againft which he was not previoufly prepared.

The other methods to be employed in ftrictures of the urethra, which occafion a permanent difficulty in voiding the urine, are, the bougies, which act mechanically. Two ways are at prefent followed of ufing thefe mechanical inftruments; the one is defigned to effect a dilatation of the paffage by flow diftenfion; the other, to deftroy the ftricture at once, by employing a greater degree of violence: only taking the precaution to keep the part dilated until the laceration is healed. The dilating bougies are conftructed of various materials, oftener fuited to the fancy of the practitioner. In the one inftance the dilatation is effected, by paffing a conical fhaped bougie through the ftricture, gradually pufhing it forward by fucceffive attempts. In the other, the bougies are ufually made cylindrical, with either a conical or a rounded point: and it often happens, that a ftricture of long continuance is removed, by paffing a firm bougie of the fize of the natural urethra, at once through the difeafed part. A great practitioner in London,* has been in the habit of employing a large filver probe for this purpofe, and, to my own knowledge, with frequent fuccefs. The impediment, which ufually conftitutes a recent ftricture, is the contraction of a flender piece of the inner membrane of the urethra, only a few lines in thicknefs, and which may be broken down by a flight mechanical force. The moft fimple method to be recommended in fuch ftrictures is, that of the bougie for the purpofe of dilatation. Thefe are made of hide leather, cut nicely, and polifhed; or of thick catgut, which fwell by abforbing moifture in the urethra, and thus enlarge the ftricture, after they have been paffed with eafe in a dry ftate.

The bougies made of plafter, fpread on linen, and rolled up, have not the abforbing property of the two former, and the wax, &c. foon becomes fo foft by the heat of the body as to render them incapable of a due degree of refiftance, Mr.

* Mr. Cruikfkank.

Mr. Smith, chemist, &c. of Tavistock-street, Covent-garden, has lately contrived a compound metal, out of which he makes bougies of all forms and sizes; the degree of flexibility of the metal, and the polish it bears, are admirable qualities for the manufacture of bougies. Where mechanical force is preferred, or the dilatation of a stricture by a conical bougie, or where the passage is so narrow as not to admit any other substance from the comparative want of resistance, these metallic bougies are, in my estimation, decidedly the best. They are also well adapted for examining the passage to ascertain the seat, &c. of stricture. For clearing the canal previously to the introduction of a caustic bougie, and for dilating the opening after a certain degree of ulceration has been excited by caustic, these instruments will be found preferable to most others.

I have the honour to be,

GENTLEMEN,

Your obedient, &c.

ANTH. CARLISLE.

*Soho-Square,*

